# Imputation-Aware Optimization of Ultra-Low-Density SNP Panels: Synthetic Benchmarking and Evaluation in a *Camellia oleifera* Family

**DOI:** 10.3390/plants15213346

**Published:** 2026-10-28

**Authors:** Yan Xu, Xiaofeng Tan

**Affiliations:** College of Forestry, Central South University of Forestry and Technology, Changsha 410004, China

**Keywords:** genomic prediction, genotype imputation, SNP panel optimization, synthetic benchmark, submodular optimization, *Camellia oleifera*

## Abstract

Ultra-low-density single-nucleotide polymorphism panels require marker selection that accounts for subsequent imputation. We formulate imputation-aware panel optimization (IAPO) by combining recoverability, training-only utility, and chromosome balance in a monotone submodular objective. We clarify the conditions linking coverage to imputation error and verify a ridge-prediction perturbation bound. Ten independent synthetic replicates, each with 720 individuals and 3000 dosage-coded loci, give a 192-marker prediction correlation of 0.347±0.092 (mean ± standard deviation). The paired advantage over coverage-only selection is 0.058 (95% confidence interval 0.010–0.106). Alternative architectures, reference-based imputation, population shifts, and candidate-ranking metrics expose important dependencies; 192 markers are not a reproducible optimum. A separate reanalysis of published *C. oleifera* data evaluates 164 offspring with normalized segregation-state genotypes and observed oil content and fruit yield. Across two repetitions of five-fold within-family validation, the 192-marker IAPO phenotype correlations are 0.067±0.112 for oil content and 0.153±0.145 for fruit yield. Prediction is modest and method rankings depend on trait and imputer. This empirical workflow test does not establish transfer across families or validate polyploid dosage calling.

## 1. Introduction

*Camellia oleifera* is a long-lived woody oil crop for which conventional phenotypic selection is intrinsically slow because economically important traits emerge over multiple seasons. Recent genomic resources have substantially changed this breeding landscape. A haplotype-resolved tetraploid genome clarified polyploid architecture and agronomic differentiation [1], while a high-density double-digest restriction site-associated DNA sequencing (ddRAD) map localized fruit- and oil-related quantitative trait loci (QTLs) on the 15 linkage groups [2]. These resources complement a broad synthesis of oiltea-camellia genomics and molecular breeding [3]. Population-genomic evidence also shows extensive single-nucleotide polymorphism (SNP) and copy-number variation associated with freezing adaptation in wild polyploid material [4]. Together, these advances make genome-enabled early selection increasingly feasible, but dense genotyping remains difficult to deploy across thousands of seedlings.

Genomic selection replaces repeated marker-by-marker testing with genome-wide prediction of genetic merit. Sparse genomic prediction shows that incomplete genotype-by-environment matrices can still support useful cultivar ranking [5]. In spring wheat, a 2026 comparison of marker platforms demonstrated that lower-density systems can remain competitive when combined with informative fixed effects [6]. More aggressively, low-density panels can be coupled with genotype imputation. GS-Impute, a genotype-imputation framework for genomic selection, reconstructs dense marker information from sparse observations for cross-population genomic selection [7], while event-driven implementations of genotype imputation emphasize that computation itself becomes a relevant design constraint at scale [8]. These findings motivate a shift from asking how many markers are affordable to asking which markers make missing genomic information most recoverable.

Marker choice is therefore an information-allocation problem rather than a simple filtering problem. Public soybean data show that prediction accuracy depends jointly on marker density and the genetic relation between training and target populations [9]. In tomato, carefully selected low-density SNP sets retained substantial prediction ability for bacterial-wilt resistance [10]. Ultra-low-coverage sequencing studies further demonstrate that imputation quality can remain high when sparse observations are informative relative to the reference panel [11]. For *C. oleifera*, recent specific-locus amplified fragment sequencing (SLAF-seq) fingerprinting [12] and ddRAD analysis of a crossing population [13] confirm abundant sequence-level diversity suitable for targeted marker development. A panel-design objective should therefore reward recoverable genome-wide information, not only minor-allele frequency, physical spacing, or marginal association.

Prediction quality also depends on how high-dimensional genomic information is modeled. Prior-data fitted networks provide a recent framework specifically developed for genomic prediction [14], while optimization of training sets has shown that data allocation affects recovery of superior candidate genotypes [15]. Genome-wide association studies remain useful for anchoring trait-relevant loci [16], but prediction frequently benefits from distributed polygenic signals. A proximal multi-objective formulation for incorporating polygenic breeding values into genomic prediction highlights the value of explicitly balancing competing genomic objectives [17]. Cropformer further shows that attention-based modeling can exploit multilocus interactions and long-range structure across crops [18]. Network-guided genomic prediction likewise demonstrates that structured genomic and multi-omics information can improve phenotype prediction [19].

The panel-design problem is also closely related to feature selection under strong redundancy. Recent Institute of Electrical and Electronics Engineers (IEEE) work on multi-scale co-expression modeling formalizes how compact genomic feature subsets can be selected while preserving predictive information [20]. Marker-assisted breeding, however, must retain biologically validated loci when they have direct operational value. Soybean QTL screening provides an example of trait-specific markers that can be fixed in a targeted assay [21], whereas perennial-crop predictive breeding emphasizes the need to integrate genomic and multi-omics information early in long breeding cycles [22]. Stress-tolerance prediction in faba bean further illustrates that prediction accuracy varies with marker composition, environment, and validation design [23]. These observations motivate a panel framework that permits mandatory loci while optimizing the residual marker budget.

Modern genomic sequence models reinforce the importance of robust validation. Progressive fine-tuning of a genome foundation model has recently shown strong supervised performance across diverse functional-genomics tasks [24]. For breeding, however, model complexity should not obscure the need for leakage-free validation and a direct connection between marker compression and downstream selection quality. Ranking-focused genomic prediction has shown that sensitivity to the highest- and lowest-ranked cultivars can differ from global prediction error [25]. Consequently, an ultra-low-density panel should be evaluated through imputation error, prediction correlation, ranking stability, robustness to reference mismatch, and genotyping cost rather than through a single marker-level score.

From a statistical perspective, our work builds on the classical genomic-selection formulation of Meuwissen, Hayes, and Goddard [26], the genomic relationship matrix developed by VanRaden [27], and ridge-regression implementations widely used in plant breeding [28]. Marker-density reduction introduces an additional transformation between observed genotypes and genomic prediction: the selected panel first generates an imputed dense representation. Panel composition therefore controls the perturbation applied to the downstream predictor. This structure makes it possible to derive a mathematical chain from marker selection to recoverability, from recoverability to imputation error, and from imputation error to prediction stability.

We propose imputation-aware panel optimization (IAPO), which unifies local linkage-disequilibrium (LD) coverage, training-only predictive utility, and chromosome-level balance. The combined objective is normalized, monotone, and submodular, so greedy construction attains the classical 1 − 1/*e* approximation guarantee under a cardinality budget. A complementary finite-sample perturbation bound propagates genotype-imputation error through ridge genomic prediction. We evaluate IAPO with a fully synthetic tetraploid-dosage benchmark motivated by the marker-budget problem in *C. oleifera*; no empirical haplotypes, pedigrees, or species-specific LD estimates are used for calibration. The simulations test information recovery and candidate ranking under controlled architectures and reference conditions. A separate reanalysis of a published *C. oleifera* full-sib family tests the executable workflow on observed marker states and field phenotypes [2]. This within-family evaluation is distinguished from polyploid dosage validation and transfer to unrelated breeding populations.

## 2. Biological and Statistical Formulation

### 2.1. Breeding Target and Dense Reference Representation

Consider a dense discovery panel with *p* biallelic SNPs scored as allele dosages in a tetraploid-dosage representation. For individual *r*, the dosage vector is $g_{r}={\left(g_{r1},\ldots ,g_{rp}\right)}^{⊤}$, where $g_{rj}\in \left\{0,1,2,3,4\right\}$. After centering and scale normalization, the dense genotype matrix is $G\in R^{n\times p}$. We use a standard additive genomic predictor as the downstream decision layer,

(1) $$y=Gb+\varepsilon ,\;E\left(\varepsilon \right)=0,\;\mathrm{Var}\left(\varepsilon \right)=\sigma_{e}^{2}I.$$

Here, $b\in R^{p}$ denotes distributed marker effects. The purpose of panel design is not to assume that all causal effects are known. Instead, it is to choose S⊆V={1,…,p} with |S|≤k so that sparse observations $G_{S}$ retain enough information to recover the untyped loci and preserve prediction.

For each locus *i*, let c(i)∈{1,…,C} denote its chromosome and let d(i,j) denote within-chromosome distance. We define a recoverability matrix $Q=[q_{ij}]$ with $0\leq q_{ij}\leq 1$ and $q_{ij}=0$ for loci on different chromosomes unless long-range information has been explicitly estimated. In a real reference population, $q_{ij}$ can be a lower confidence bound on cross-validated dosage $R^{2}$ when locus *j* is imputed from typed locus *i* or from a local typed neighborhood. In this benchmark, $q_{ij}={\mathrm{Cor}}_{\mathrm{train}}{\left(g_{i},g_{j}\right)}^{2}$ within chromosomes, with $q_{ii}=1$, and $w_{j}=\eta_{c}=1$. These empirical scores are optimization proxies, not conservative population lower bounds. They are re-estimated from each replicate’s training genotypes.

### 2.2. Panel Design as Information Allocation

A conventional uniform panel allocates markers by physical spacing, whereas a minor-allele-frequency (MAF)-based panel prioritizes loci with high heterozygosity. Both strategies can be useful, but neither directly measures the information recoverable at untyped loci. We therefore define a coverage term

(2) $$C\left(S\right)=\sum_{j=1}^{p}w_{j}\max_{i\in S}q_{ij},$$

with non-negative locus weights $w_{j}$. When S=⌀, the maximum is defined as zero. Equation (2) is a facility-location form: a selected marker receives credit when it offers a better imputation anchor for a dense locus than markers already in the panel. This avoids repeatedly rewarding redundant SNPs from the same strong LD block.

Coverage alone can still overlook trait-relevant or structurally informative loci. Let $a_{i}$ be a training-only marginal association score, $\ell_{i}$ a principal component analysis (PCA) leverage score, and $h_{i}$ a heterozygosity score, all rescaled to [0,1]. We define

(3) $$u_{i}=\omega_{a}a_{i}+\omega_{\ell }\ell_{i}+\omega_{h}h_{i},\;\omega_{a}+\omega_{\ell }+\omega_{h}=1,$$

and the modular predictive term

(4) $$U\left(S\right)=\sum_{i\in S}u_{i}.$$

Only training individuals are used to compute $a_{i}$ and $\ell_{i}$. Validation and test phenotypes therefore remain isolated from panel optimization. Specifically, let *Z* have training-standardized columns and let $Z=UDV^{⊤}$ be its singular value decomposition. Before normalization, the three scores are $\left|\mathrm{Cor}(g_{i},y)\right|$, $\sum_{h=1}^{10}V_{ih}^{2}$, and $2{\hat{f}}_{i}\left(1-{\hat{f}}_{i}\right)$, where ${\hat{f}}_{i}={\bar{g}}_{i}/4$. Each score vector is transformed by $\left(v_{i}-\min v\right)/\left(\max v-\min v\right)$; a vector with range at most $10^{-12}$ maps to zero. The utility weights are (0.45,0.30,0.25). The heterozygosity term is an allele-frequency diversity proxy, not the probability of a heterozygous tetraploid genotype. Correlated components are not decorrelated or interpreted as independent evidence; their overlap can amplify a signal, motivating the weight sensitivity analysis.

### 2.3. Chromosome Balance

An ultra-low-density panel can collapse onto chromosomes with long LD or strong training-set signals. For a perennial breeding panel, such concentration is undesirable because reference populations change over cycles and future traits may rely on genomic regions not emphasized by the current training phenotype. To regularize coverage across the 15 chromosomes, let

(5) $$B\left(S\right)=\sum_{c=1}^{C}\eta_{c}\log\;\left(1+n_{c}\left(S\right)\right),\;n_{c}\left(S\right)=\sum_{i\in S}1\left\{c\left(i\right)=c\right\}.$$

Here, $\eta_{c}\geq 0$ may reflect chromosome length or target importance. The logarithm produces diminishing returns: adding the first few markers to an underrepresented chromosome is valuable, whereas repeatedly adding markers to an already dense chromosome yields a smaller balance gain.

**Remark** **1.** *The formulation separates three biological roles that are often conflated in low-density panel design. C(S) preserves imputability of the dense genome, U(S) protects training-supported predictive signal, and B(S) prevents the budget from collapsing into a few favorable LD blocks. Because all three terms are interpretable, their relative weights can be audited and changed when a breeding program moves to a new population, trait, or genotyping platform.*

## 3. Imputation-Aware Panel Optimization

### 3.1. Unified Objective

The IAPO objective is

(6) F(S)=αC(S)+βU(S)+γB(S),S⊆V,|S|≤k,

where α,β,γ≥0. The weights determine how strongly the design favors recoverability, training-supported utility, and broad chromosomal representation. In the benchmark, α=1, β=3.5, and γ=2. These are prespecified heuristic defaults, not a grid-search optimum; they are not retuned on the test set. The sensitivity analysis halves or doubles β and separately removes γ, without choosing a winner from test outcomes. Robustness across traits is an empirical question.

**Figure 1:**
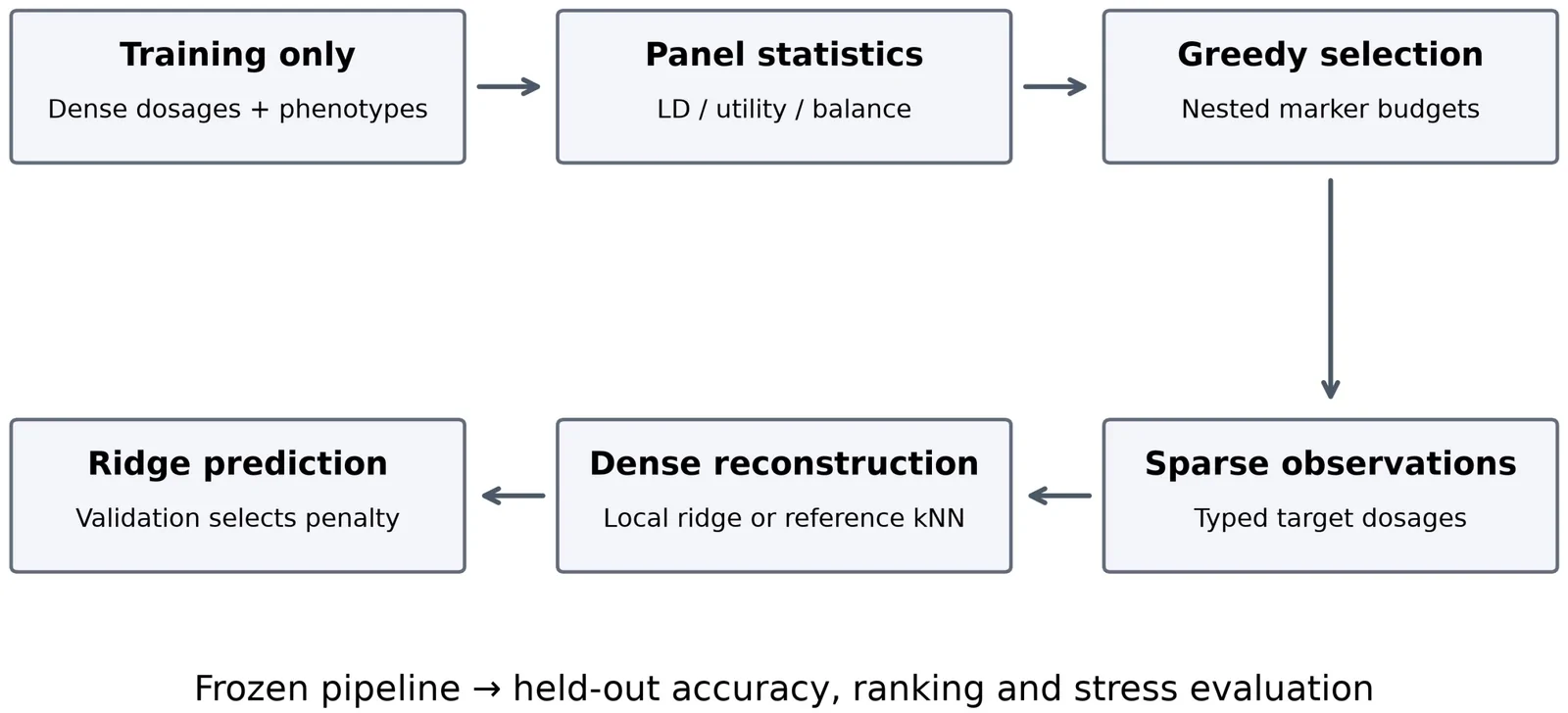
Conceptual workflow of imputation-aware panel optimization.

Figure 1 summarizes the information flow. Dense reference genotypes are used only during panel development. LD/recoverability statistics and training-only marker utility feed the IAPO selector. Future individuals are genotyped only at the selected SNPs, after which local dosage imputation reconstructs a dense representation for genomic prediction. This architecture keeps the design objective aligned with the actual deployment pathway: sparse assay, imputation, prediction, and early selection.

Figure 1 also emphasizes two safeguards. First, panel construction is restricted to reference genotypes and training phenotypes, so validation and test outcomes cannot leak into marker choice. Second, the final evaluation is separate from the training and validation stages; no feedback from the test outcomes is used for panel or penalty selection. The method is therefore deployable as an iterative breeding workflow: periodically refresh the reference panel, re-estimate recoverability, redesign only when information drift is material, and retain the same downstream genomic-prediction interface.

### 3.2. Submodularity and Greedy Construction

For A⊆B⊆V and e∉B, define the marginal gain $\Delta_{F}\left(e|A\right)=F\left(A\cup \left\{e\right\}\right)-F\left(A\right)$. Submodularity requires diminishing returns,

(7) $$\Delta_{F}\left(e|A\right)\geq \Delta_{F}\left(e|B\right).$$

This property is valuable because the exact cardinality-constrained maximum of a general set function is combinatorial, whereas monotone submodularity supports an explicit approximation guarantee.

**Theorem 1** (Monotone submodularity of IAPO)**.** *Assume $w_{j}\geq 0$, $q_{ij}\in \left[0,1\right]$, $u_{i}\geq 0$, $\eta_{c}\geq 0$, and α,β,γ≥0. Then, F in Equation (6) is normalized, monotone, and submodular.*

**Proof.**  Normalization follows because C(⌀)=U(⌀)=B(⌀)=0. For each dense locus *j*, define

(8) $$f_{j}\left(S\right)=w_{j}\max_{i\in S}q_{ij},\;f_{j}\left(⌀\right)=0.$$

For A⊆B and e∉B, write $m_{A}=\max_{i\in A}q_{ij}$ and $m_{B}=\max_{i\in B}q_{ij}$. Since $m_{A}\leq m_{B}$,

(9) $$\begin{array}{cc} \Delta f_{j}\left(e|A\right) & =w_{j}\max\left\{q_{ej}-m_{A},0\right\}\\ \; & \geq w_{j}\max\left\{q_{ej}-m_{B},0\right\}=\Delta f_{j}\left(e|B\right). \end{array}$$

Hence, every $f_{j}$ is submodular and monotone, and so is $C\left(S\right)=\sum_{j}f_{j}\left(S\right)$. The utility term is modular because

(10) $$\Delta U\left(e|A\right)=u_{e}=\Delta U\left(e|B\right),$$

so it is both submodular and supermodular. For chromosome balance, only chromosome c(e) changes when *e* is added. Therefore,

(11) $$\Delta B\left(e|S\right)=\eta_{c(e)}\;\left[\log\;\left(2+n_{c(e)}\left(S\right)\right)-\log\;\left(1+n_{c(e)}\left(S\right)\right)\right].$$

The bracketed increment decreases as $n_{c(e)}\left(S\right)$ increases because log(1+x) is concave. Since A⊆B implies $n_{c(e)}\left(A\right)\leq n_{c(e)}\left(B\right)$, we obtain ΔB(e∣A)≥ΔB(e∣B). Every marginal gain is non-negative. Thus, *B* is monotone submodular. A non-negative linear combination of normalized monotone submodular functions is again normalized, monotone, and submodular, proving the claim. □

The panel can therefore be built greedily, using the classical cardinality guarantee for monotone submodular maximization [29]. Starting with $S_{0}=⌀$, choose

(12) $$e_{t}\in \arg\max_{e\in V\backslash S_{t}}\Delta_{F}\left(e|S_{t}\right),\;S_{t+1}=S_{t}\cup \left\{e_{t}\right\},$$

until $|S_{k}|=k$. The supplied implementation updates all marginal gains on the affected chromosome after every addition; gains on other chromosomes are unchanged. This gives the exact greedy sequence with marker-index tie-breaking. Table 1 describes an equivalent lazy priority-queue implementation for larger problems.

**Theorem 2** (Approximation guarantee)**.** *Let $S^{★}\in \arg\max_{|S|\leq k}F\left(S\right)$ and let $S_{k}$ be generated by Equation (12). Then,*

(13) $$F\left(S_{k}\right)\geq \left[1-{\left(1-\frac{1}{k}\right)}^{k}\right]F\left(S^{★}\right)\geq \left(1-e^{-1}\right)F\left(S^{★}\right).$$

**Proof.**  At iteration *t*, submodularity and monotonicity imply

(14) $$F\left(S^{★}\right)-F\left(S_{t}\right)\leq F\left(S_{t}\cup S^{★}\right)-F\left(S_{t}\right)\leq \sum_{e\in S^{★}\backslash S_{t}}\Delta_{F}\left(e|S_{t}\right).$$

There are at most *k* elements in $S^{★}\backslash S_{t}$. Hence, at least one has marginal gain no smaller than the average residual gap divided by *k*. Because greedy chooses the largest marginal gain,

(15) $$F\left(S_{t+1}\right)-F\left(S_{t}\right)\geq \frac{F\left(S^{★}\right)-F\left(S_{t}\right)}{k}.$$

Rearranging yields

(16) $$F\left(S^{★}\right)-F\left(S_{t+1}\right)\leq \left(1-\frac{1}{k}\right)\left[F\left(S^{★}\right)-F\left(S_{t}\right)\right].$$

Applying this recursion from $S_{0}=⌀$ gives

(17) $$F\left(S^{★}\right)-F\left(S_{k}\right)\leq {\left(1-\frac{1}{k}\right)}^{k}F\left(S^{★}\right),$$

which proves the first inequality. The second follows from ${\left(1-1/k\right)}^{k}\leq e^{-1}$. □

### 3.3. Connection to Imputation Error

The coverage term has a direct statistical interpretation when $q_{ij}$ is a conservative lower bound on local imputation $R^{2}$. Let $G_{j}$ be a dosage variable at dense locus *j* and let ${\hat{G}}_{j}\left(S\right)$ be its population least-squares linear projection, including an intercept, onto an admissible predictor space. The space must include each selected anchor contributing to the maximum below. This population projection is distinct from a fitted, regularized local imputer.

**Proposition 1** (Coverage-controlled imputation error)**.** *Suppose, for each dense locus j, the achievable local linear-imputation coefficient of determination satisfies*

(18) $$R_{j}^{2}\left(S\right)\geq \max_{i\in S}q_{ij}.$$

*Then, the weighted mean-squared imputation error satisfies*

(19) $$\sum_{j=1}^{p}w_{j}E\;\left[{\left(G_{j}-{\hat{G}}_{j}\left(S\right)\right)}^{2}\right]\leq \sum_{j=1}^{p}w_{j}\sigma_{j}^{2}\left(1-\max_{i\in S}q_{ij}\right),$$

*where $\sigma_{j}^{2}=\mathrm{Var}\left(G_{j}\right)$.*

**Proof.**  For the least-squares linear predictor of $G_{j}$ from its selected local predictors, the residual variance is

(20) $$E\;\left[{\left(G_{j}-{\hat{G}}_{j}\left(S\right)\right)}^{2}\right]=\sigma_{j}^{2}\left\{1-R_{j}^{2}\left(S\right)\right\}.$$

By assumption (18), $1-R_{j}^{2}\left(S\right)\leq 1-\max_{i\in S}q_{ij}$. Multiplying by the non-negative weight $w_{j}$ and summing over all dense loci gives Equation (19). Thus, increasing facility-location coverage decreases this upper envelope only when the stated calibration and predictor-space assumptions hold. Sample correlations, finite-reference estimation, restricted nearest-neighbor models, and target distribution shifts do not automatically satisfy them. □

We therefore diagnose rather than presume Equation (18). For each untyped test locus, using mean-squared error (MSE), we compare $1-{\mathrm{MSE}}_{j,\mathrm{test}}/s_{j,\mathrm{train}}^{2}$ with $\max_{i\in S}q_{ij}$. This quantity can be negative and is not a squared Pearson correlation. The fraction below the proxy measures empirical violations of the proposed inequality, not a significance test. Section 6 reports both locus-level violations and aggregate error; the theorem is not used as an empirical certificate for the implemented imputer.

### 3.4. Nested Budgets, Mandatory Markers, and Computational Complexity

A practical breeding program rarely develops only one assay size. It is often preferable to construct a nested family of panels so that a 48-SNP pilot can be expanded to 96, 192, or 384 SNPs without discarding validated loci. The greedy sequence in Equation (12) provides this property automatically when it is run once to the largest budget and intermediate panels are defined by prefixes of the selection order.

**Lemma 1** (Nested greedy panels)**.** *Let $\left(e_{1},\ldots ,e_{K}\right)$ be a greedy sequence for a fixed objective F and candidate set V. Define $S_{k}=\left\{e_{1},\ldots ,e_{k}\right\}$ for 1≤k≤K. Then, $S_{k_{1}}\subseteq S_{k_{2}}$ whenever $k_{1}\leq k_{2}$, and every $S_{k}$ separately satisfies the approximation bound in Equation (13) for its own cardinality budget k.*

**Proof.**  The nesting statement follows directly from the prefix definition. For any fixed *k*, the first *k* choices in the common sequence are exactly the choices that the greedy algorithm would make if it stopped at budget *k*, because the marginal-gain rule at iteration t<k does not depend on the future stopping budget. The proof of Theorem 2 can therefore be applied independently to each prefix $S_{k}$, giving the same cardinality-specific approximation factor. □

Breeding programs may also require a set *M* of compulsory loci, for example, validated markers linked to oil composition, flowering, disease resistance, or identity control. Let the remaining budget be k−|M| and define the conditioned objective

(21) $$F_{M}\left(S\right)=F\left(M\cup S\right)-F\left(M\right),\;S\subseteq V\backslash M.$$

This representation allows for marker-assisted selection constraints to be integrated without changing the structure of the optimization problem.

**Proposition 2** (Conditioning on mandatory markers)**.** *If F is normalized, monotone, and submodular, then $F_{M}$ in Equation (21) is normalized, monotone, and submodular on V\M. Greedy selection of the residual k−|M| markers therefore retains the standard 1−1/e approximation guarantee relative to the best residual augmentation of M.*

**Proof.**  Normalization is immediate because $F_{M}\left(⌀\right)=F\left(M\right)-F\left(M\right)=0$. For A⊆B⊆V\M and e∉B, monotonicity of *F* gives F(M∪A∪{e})≥F(M∪A). Moreover,

(22) $$\begin{array}{cc} \Delta F_{M}\left(e|A\right) & =F(M\cup A\cup \{e\})-F(M\cup A)\\ \; & \geq F(M\cup B\cup \{e\})-F(M\cup B)\\ \; & =\Delta F_{M}\left(e|B\right). \end{array}$$

The inequality is exactly the diminishing-returns property of *F* because M∪A⊆M∪B. Thus, $F_{M}$ is monotone submodular. Applying Theorem 2 with residual budget k−|M| completes the proof. □

For dense *Q*, direct greedy evaluation has worst-case cost O(k|V|p) because each candidate can update coverage over *p* loci at every iteration. Block storage omits the cross-chromosome zeros without changing the objective. Dropping positive within-chromosome scores instead defines a different, sparse objective and can change the selected panel; no such thresholding is used here. Lazy greedy evaluation exploits submodularity: a previously computed marginal gain is an upper bound on its future value. With consistent tie-breaking, lazy evaluation preserves the exact greedy sequence for the same fixed *Q*. The supplied code instead updates all gains on the affected chromosome, also exactly.

**Remark** **2.** *The approximation guarantee concerns the explicit panel-design objective, not genomic-prediction correlation itself. This distinction is important. The theorem guarantees that the selected panel is near-optimal for the calibrated combination of recoverability, utility, and chromosome balance. Prediction is then evaluated independently on held-out individuals. Nested prefixes and mandatory-marker conditioning make this guarantee compatible with realistic assay expansion and marker-assisted breeding constraints rather than restricting it to a single unconstrained panel.*

## 4. Stability of Genomic Prediction Under Imputation Error

### 4.1. Ridge Genomic Predictor

Let $X\in R^{n\times p}$ be the standardized dense genotype matrix in the training sample and let $\tilde{X}=X+E$ be its imputed counterpart. Ridge genomic prediction estimates

(23) $$\hat{b}={\left(X^{⊤}X+\lambda I\right)}^{-1}X^{⊤}y,\;\lambda >0,$$

and

(24) $$\tilde{b}={\left({\tilde{X}}^{⊤}\tilde{X}+\lambda I\right)}^{-1}{\tilde{X}}^{⊤}y.$$

The following result makes the error-propagation mechanism explicit.

**Theorem 3** (Finite-sample ridge perturbation bound)**.** *Let $A=X^{⊤}X+\lambda I$ and $\tilde{A}={\tilde{X}}^{⊤}\tilde{X}+\lambda I$. Then,*

(25) $$∥\tilde{b}-\hat{b}{∥}_{2}\leq {∥y∥}_{2}\left[\frac{{∥E∥}_{2}}{\lambda }+\frac{\left({2∥X∥}_{2}{∥E∥}_{2}+{∥E∥}_{2}^{2}\right){∥X∥}_{2}}{\lambda^{2}}\right].$$

*For a test genotype x with imputation error e and $\tilde{x}=x+e$,*

(26) $$|{\tilde{x}}^{⊤}\tilde{b}-x^{⊤}\hat{b}{|\leq ∥x∥}_{2}∥\tilde{b}-\hat{b}{∥}_{2}+{∥e∥}_{2}\left(∥\hat{b}{∥}_{2}+{∥\tilde{b}-\hat{b}∥}_{2}\right).$$

**Proof.**  Because $X^{⊤}X$ and ${\tilde{X}}^{⊤}\tilde{X}$ are positive semidefinite, $∥A^{-1}{∥}_{2}\leq 1/\lambda$ and $∥{\tilde{A}}^{-1}{∥}_{2}\leq 1/\lambda$. Expanding the perturbed normal matrix gives

(27) $$\tilde{A}=A+\Delta_{A},\;\Delta_{A}=X^{⊤}E+E^{⊤}X+E^{⊤}E.$$

The resolvent identity yields

(28) $${\tilde{A}}^{-1}-A^{-1}=-{\tilde{A}}^{-1}\Delta_{A}A^{-1}.$$

Hence,

(29) $$\begin{array}{cc} \tilde{b}-\hat{b} & ={\tilde{A}}^{-1}{\left(X+E\right)}^{⊤}y-A^{-1}X^{⊤}y\\ \; & ={\tilde{A}}^{-1}E^{⊤}y-{\tilde{A}}^{-1}\Delta_{A}A^{-1}X^{⊤}y. \end{array}$$

Taking operator norms and applying submultiplicativity gives

(30) $$∥\tilde{b}-\hat{b}{∥}_{2}\leq \frac{{∥E∥}_{2}{∥y∥}_{2}}{\lambda }+\frac{∥\Delta_{A}{∥}_{2}{∥X∥}_{2}{∥y∥}_{2}}{\lambda^{2}}.$$

Furthermore,

(31) $$\begin{array}{cc} ∥\Delta_{A}{∥}_{2} & \leq ∥X^{⊤}{E∥}_{2}+∥E^{⊤}{X∥}_{2}+{∥E^{⊤}E∥}_{2}\\ \; & \leq {2∥X∥}_{2}{∥E∥}_{2}+{∥E∥}_{2}^{2}. \end{array}$$

This proves Equation (25). For a test individual,

(32) $$\begin{array}{cc} {\tilde{x}}^{⊤}\tilde{b}-x^{⊤}\hat{b} & =x^{⊤}\left(\tilde{b}-\hat{b}\right)+e^{⊤}\tilde{b}\\ \; & =x^{⊤}\left(\tilde{b}-\hat{b}\right)+e^{⊤}\hat{b}+e^{⊤}\left(\tilde{b}-\hat{b}\right). \end{array}$$

The Cauchy–Schwarz inequality applied to the three terms gives Equation (26). In particular, writing $\Delta b=\tilde{b}-\hat{b}$, the exact expansion is $x^{⊤}\Delta b+e^{⊤}\hat{b}+e^{⊤}\Delta b$; the final cross term must be retained. Both fits use the same positive λ, response vector, and coordinate system. Separately tuned fits at different penalties are not covered by this comparison. □

**Corollary 1** (First-order stability)**.** *If ${∥E∥}_{2}\leq \delta$ and δ is small relative to $\lambda /{∥X∥}_{2}$, then*

(33) $${∥\tilde{b}-\hat{b}∥}_{2}=O\left(\delta \right),\;|{\tilde{x}}^{⊤}\tilde{b}-x^{⊤}\hat{b}|=O\left(\delta +{∥e∥}_{2}\right).$$

**Proof.**  In Equation (25), the terms involving ${∥E∥}_{2}$ are linear and quadratic in δ. As δ→0, the quadratic term is dominated by the linear term. Substituting the resulting O(δ) coefficient perturbation into Equation (26) gives the stated first-order prediction stability. □

To verify the algebra separately from biological performance, we generate 30 independent Gaussian design matrices of size 60×90. For perturbation scales 0, 0.01, 0.05, 0.1, and 0.2 as well as penalties cycling through 5,40,600, the script checks the exact prediction identity, coefficient bound, and both prediction envelopes. All 150 cases pass to numerical tolerance $10^{-9}$. This finite check supports implementation consistency; it does not replace the proof.

**Figure 2:**
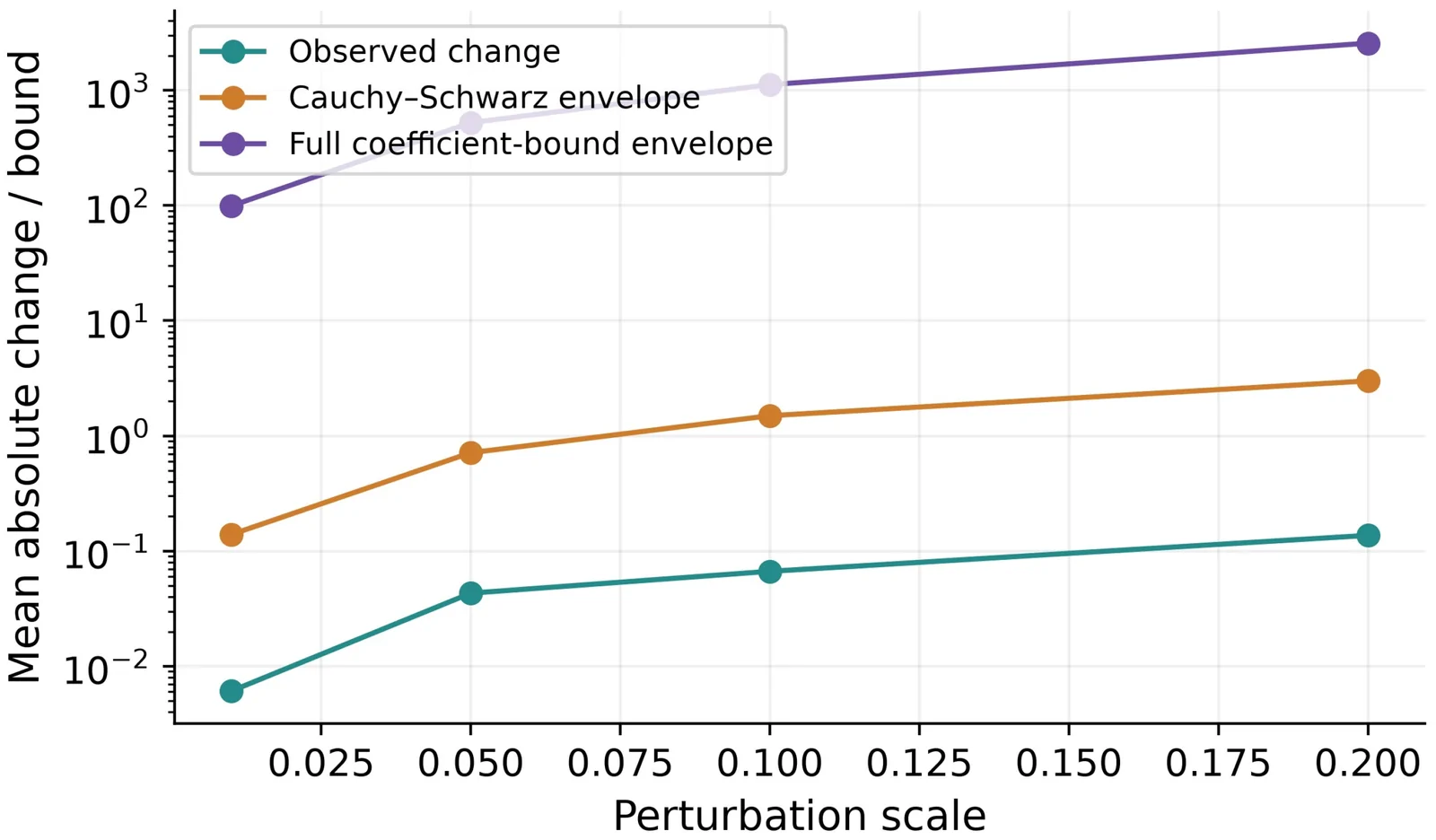
Numerical ridge-perturbation check. Curves show means over 30 Gaussian designs; zero perturbation is omitted on the logarithmic axis. The full envelope substitutes the coefficient bound into the prediction inequality.

Figure 2 shows that the bounds remain conservative. The sizable gap is expected: the Cauchy–Schwarz inequality ignores cancellation across marker effects and therefore prioritizes validity over tightness. Its role is not to predict the empirical curve, but to establish a deterministic comparison between two ridge fits under specified perturbations. No distribution-free confidence interval or guarantee of empirical panel superiority follows.

### 4.2. Robust Multi-Reference Extension

Suppose recoverability is estimated under *H* plausible reference scenarios, with $F_{h}\left(S\right)$ denoting the IAPO objective for scenario *h* and weights $\pi_{h}\geq 0$, $\sum_{h=1}^{H}\pi_{h}=1$. Define

(34) $$F_{\mathrm{rob}}\left(S\right)=\sum_{h=1}^{H}\pi_{h}F_{h}\left(S\right).$$

Because non-negative sums preserve monotone submodularity, $F_{\mathrm{rob}}$ has the same greedy approximation guarantee. This extension is useful when a breeding program anticipates multiple families, locations, or reference updates.

**Remark** **3.** *The perturbation analysis explains why an imputation-aware panel can improve prediction even when two panels have similar typed-marker counts. The relevant quantity is the error matrix induced after dense reconstruction, not the number of assayed loci alone. Ridge regularization attenuates this perturbation through λ, but cannot remove information that was never captured. Panel design and predictor regularization therefore act as complementary, rather than interchangeable, controls on genomic-prediction stability.*

## 5. Evaluation Design

### 5.1. Benchmark Construction and Data Partitioning

The experiments use a fully synthetic benchmark to isolate statistical effects of sparse dosage reconstruction. It is not calibrated to an observed *C. oleifera* population and does not represent simulated individuals as field observations. The benchmark contains 720 tetraploid individuals, 15 chromosomes, and 3000 biallelic SNPs. For each chromosome, four independent homolog chains are generated, each with within-chain Markov persistence $\rho_{c}=0.80+0.015\left[\left(c-1\right)\;\mathrm{mod}\;7\right]$, and their sum gives allele dosage in {0,1,2,3,4}. At a new chain position, the previous allele is retained with probability $\rho_{c}$; otherwise, it is redrawn with probability $f_{cj}$, where $f_{cj}$ is drawn from a Beta(1.5,3) distribution and clipped to [0.05,0.45]. These are refresh probabilities; persistence means that the realized marginal allele frequencies need not equal them. Summing four chains reproduces dosage support and local correlation, but not meiosis, double reduction, or preferential pairing.

Trait architecture is deliberately polygenic. Seventy-two additive QTLs are distributed across the genome and 18 weak pairwise interactions introduce modest non-additive structure. With standardized dosage $z_{rj}$, additive effect $a_{j}$, and interaction coefficient $\kappa_{j\ell }$, the latent genetic signal for individual *r* is

(35) $$g_{r}^{★}=\sum_{j\in Q}z_{rj}a_{j}+\sum_{(j,\ell )\in P}\kappa_{j\ell }z_{rj}z_{r\ell }.$$

Independent noise is scaled so that the benchmark genetic-signal variance fraction is 0.55, with expected noise variance 0.45/0.55 relative to the training-standardized latent signal; the realized fraction fluctuates. Phenotypes and causal-marker standardization use training moments. A fresh random partition is created before panel construction in every replicate: 480 individuals are used for panel statistics and model fitting, 120 for tuning, and 120 remain untouched until final test evaluation.

Table 2 records the benchmark quantities required for regeneration. The 3000-SNP dense representation is intentionally moderate: it preserves chromosome-scale redundancy while allowing for all panel budgets to be evaluated reproducibly on a standard workstation. Genotypes, marker positions, QTL indicators, epistatic pairs, phenotypes, and the replicate splits are stored explicitly in the accompanying archive. No test phenotype is used to select markers, fit local imputation models, or tune the genomic predictor.

### 5.2. Panel Construction, Local Imputation, and Evaluation Metrics

Four strategies are compared under identical budgets of 48, 96, 192, and 384 SNPs. Uniform uses chromosome-wise near-regular spacing. MAF prioritizes training minor-allele frequency, using marker-index tie-breaking and no spacing constraint. LD-Greedy optimizes only the recoverability term C(S). IAPO optimizes the complete objective F(S) with α=1, β=3.5, and γ=2. Its training-only utility combines marginal phenotype association, PCA leverage, and heterozygosity with fixed weights 0.45, 0.30, and 0.25. IAPO and LD-Greedy are nested greedy sequences, so larger budgets extend rather than replace smaller panels.

For each untyped locus *j*, $N_{j}\left(S\right)$ contains the nearest typed marker on every available side within the same chromosome. Let $X_{j}$ be the centered training design matrix for those typed neighbors and $y_{j}$ the centered training dosage vector at locus *j*. Local ridge reconstruction uses τ=0.8:

(36) $${\hat{g}}_{rj}={\bar{g}}_{j}+{\left(g_{r,N_{j}\left(S\right)}-{\bar{g}}_{N_{j}\left(S\right)}\right)}^{⊤}{\left(X_{j}^{⊤}X_{j}+\tau I\right)}^{-1}X_{j}^{⊤}y_{j}.$$

Predicted dosages are clipped to [0,4]. Each reconstructed marker is standardized using training-set moments only. The downstream ridge predictor is fit on the training set, while the regularization parameter is selected from {5,15,40,100,250,600} using the validation set. Penalty tuning minimizes validation mean-squared error. Test outcomes are used only after the corresponding pipeline is frozen. The 192-marker setting is a prespecified diagnostic budget, not selected using its test performance.

Primary outcomes are dosage-imputation correlation, normalized root-mean-squared error (NRMSE), test prediction correlation, and prediction root-mean-squared error (RMSE). To compare density without claiming vendor-specific prices, we use the normalized cost index

(37) $$\mathrm{Cost}\left(k\right)=0.18+0.82\frac{k}{384}.$$

The index is used only to expose within-benchmark accuracy–cost trade-offs; it is not an empirical procurement price.

Metric interpretation is fixed across all budgets. Imputation correlation pools training-standardized dosages over untyped loci only; typed loci are excluded. Dosage NRMSE is the raw untyped-locus RMSE divided by the dosage range, four. Prediction correlation and RMSE use held-out phenotypes standardized by training moments. The first pair therefore measures recovery of dense genotype information, and the second pair measures downstream selection utility. Uniform tests physical spacing, MAF tests allele-frequency enrichment, LD-Greedy tests recoverability alone, and IAPO additionally uses training-supported utility and chromosome balance. Thus, the LD-Greedy versus IAPO comparison assesses the combined utility and balance terms under identical marker budgets; it does not separately identify each term’s contribution. The same definitions are retained in Section 5.3.

A second, reference-based imputer uses the five nearest training individuals within each chromosome. Typed dosages are divided by training standard deviations (floored at 0.1), and squared Euclidean distances are then computed; weights are proportional to $1/(d+10^{-6})$. Untyped dosages are weighted reference means and typed values are copied. Training individuals exclude themselves when reconstructed. With no typed marker on a chromosome, both engines use training means. This nearest-neighbor method tests a different reconstruction mechanism, but is not a phased haplotype or pedigree model.

We additionally report Spearman’s rank correlations and top-5%/top-10% overlap against both phenotype and the known simulated genetic signal. For 120 test individuals, these sets contain six and twelve candidates; overlap is intersection size divided by set size, with marker-independent individual-index tie-breaking. The latent signal includes interactions and is not a validated additive breeding value. All summary tables report mean ± sample standard deviation (SD) across ten seeds; paired 95% confidence intervals (CIs) for differences use $\bar{d}\pm t_{9,0.975}s_{d}/\sqrt{10}$. These intervals describe simulation uncertainty, are exploratory, and are not adjusted for multiple comparisons.

### 5.3. Reproducibility, Leakage Control, and Robustness Protocol

The computational workflow now regenerates all numerical results from simulated inputs, rather than merely plotting historical result tables. Each of ten seeds redraws genotypes, causal effects, interactions, noise, and partitions. The archive stores individual predictions, selected panels, replicate metrics, summaries, and executable verification scripts. Alternative architectures use 12 additive QTLs, 300 additive QTLs, or an interaction coefficient of 0.8 rather than 0.08. A short-LD reference condition reduces all persistence values by 0.15 for both reference and target and re-estimates panel scores. Each condition has ten replicate runs; architecture comparisons share seed indices.

Leakage prevention is enforced at every stage. Marker association and PCA leverage are calculated on the 480 training individuals only. Local imputation coefficients are fitted only from training genotypes. The 120 validation individuals are used solely to choose ridge regularization. Dense genotype and phenotype transformations are fitted on the training partition. Untyped target dosages are used only for evaluation; a masking-invariance check verifies that modifying them does not change either imputer’s predictions. The 120 test individuals contribute neither phenotypes nor tuning information before final evaluation. Reported test performance therefore corresponds to a genuine held-out prediction step rather than repeated optimization against the final endpoint.

Budget comparability is controlled by using the same four marker counts for every strategy. For IAPO and LD-Greedy, panels are prefixes of one greedy sequence, so the 48-SNP panel is contained in the 96-, 192-, and 384-SNP versions. This nested construction makes density effects interpretable because performance changes arise from adding markers instead of swapping to unrelated assays. All panel statistics are frozen before test evaluation, and the archive records the numerical summaries used for each reported table and figure.

Robustness at 192 markers uses distinct generated targets: an independently drawn matched population; a related target choosing one training donor per target individual and independently copying each whole chromosome from that same donor with probability 0.5; a target with refresh probabilities shifted by +0.10; and a target with persistence reduced by 0.20. A further target has independent ±1 dosage errors with probability 0.10, clipped to [0,4], and is compared with its own error-free control. These are artificial sensitivity conditions, not pedigree relationships or field-calibrated drift rates. Reference-fitted scores and coefficients remain frozen within each target comparison; the short-LD reference experiment separately tests redesign after reference change.

**Remark** **4.** *This benchmark is deliberately transparent about the boundary between method testing and biological validation. It represents chromosome grouping, tetraploid dosage, heterogeneous correlation, polygenic architecture, and a held-out workflow, but it does not model a breeding population’s inheritance mechanism. Repeated splits, nested panels, executable files, and explicit stress states are intended to make later replacement by real C. oleifera reference populations straightforward.*

### 5.4. Evaluation Using a Published C. oleifera Family

We reanalyzed the open supplementary data of Lin et al. [2], using Table S1 for phenotypes and Table S4 for genotypes. The source study genotyped 180 F_1_ offspring of CL53 (female) × CL81 (male) and measured fruit and oil traits in 2015–2017 at one planting site. Sample identifiers were joined explicitly; spreadsheet row order was not assumed to match. Oil content (OC, percent of oil weight relative to dry kernel weight) was the primary endpoint, and fruit yield (FY, kg per tree) was a complementary endpoint. Each endpoint was the mean of its available annual observations, requiring at least two years for both traits. This retained 164 offspring and excluded 16 with insufficient observations. Phenotypes were not imputed. Eligibility can favor offspring with measurable fruit and oil traits, which limits the target population represented by the analysis.

The source records segregation classes, not ploidy-resolved allele dosages. We encoded ll/lm and nn/np as 0/1 and hh/hk/kk as 0/0.5/1. Three multiallelic ef×eg markers were excluded, leaving 5769 biallelic markers on 15 chromosomes. The normalized states share a numerical range but do not have a common allele-copy interpretation. Missing calls, 4.54% of entries before sample eligibility filtering, remained missing until training-based replacement. Within each fitting partition, markers required at least 90% observed calls and nonzero observed variance. Missing fitting calls and missing typed validation/test calls used that partition’s marker means. Only originally observed, untyped test entries contributed to reconstruction metrics. Physical coordinates were parsed from the published marker identifiers. The source dataset was itself filtered for linkage mapping; this ascertainment was inherited, and cannot be removed by our subsequent training-only filtering.

Two seeded repetitions of five-fold cross-validation held out each eligible tree once per repetition. Within the other four folds, 75% of trees, rounded down, formed the fitting partition and the remainder formed a separate validation partition. All annual records from one tree remained in the same partition. Thus, each run used 98 or 99 fitting trees, 33 validation trees, and 32 or 33 test trees. Marker filtering, squared-correlation recoverability, PCA leverage, association scores, imputation coefficients, and normalization used fitting trees only. The same split was used for both traits and all methods. Validation phenotypes selected the ridge penalty from the original grid; fitting and validation trees were not pooled for a later refit. Test phenotypes were used only for evaluation.

We retained the original IAPO weights, marker budgets, and local-ridge penalty without retuning them to the empirical outcomes. The implementation accepts variable chromosome sizes. Because allele frequencies are not identified by the normalized segregation coding, the MAF comparator was replaced by a state-diversity comparator, ranked by $2{\bar{x}}_{j}\left(1-{\bar{x}}_{j}\right)$; the same normalized state-diversity term replaced heterozygosity in the IAPO utility. Uniform selection allocated each chromosome a marker count proportional to its eligible marker count and chose unique markers nearest equally spaced physical positions. LD-Greedy optimized coverage only. Reconstruction used the nearest available typed marker on either physical side and was clipped to [0,1]. Dense-marker ridge prediction provided a separate comparator. At 192 markers, reference-based five-neighbor imputation tested engine sensitivity, with self-neighbors excluded in fitting reconstruction. No empirical dosage uncertainty or polyploid inheritance model was fitted.

We report test phenotype correlation, training-standardized phenotype RMSE, Spearman’s correlation, top-decile overlap, pooled training-standardized correlation of observed untyped states, and state RMSE on the [0,1] scale. Fold means and SDs summarize split sensitivity; overlapping folds are not independent biological replicates, so no independent-population confidence intervals or significance tests are assigned to these averages. This design assesses prediction among offspring from one family, not prediction across families, sites, or future years. The archive preserves the original source ZIP, its checksum, the explicit sample join, excluded records, all splits, selected panels, and individual predictions.

## 6. Results

### 6.1. Panel Placement and Imputation Accuracy

**Figure 3:**
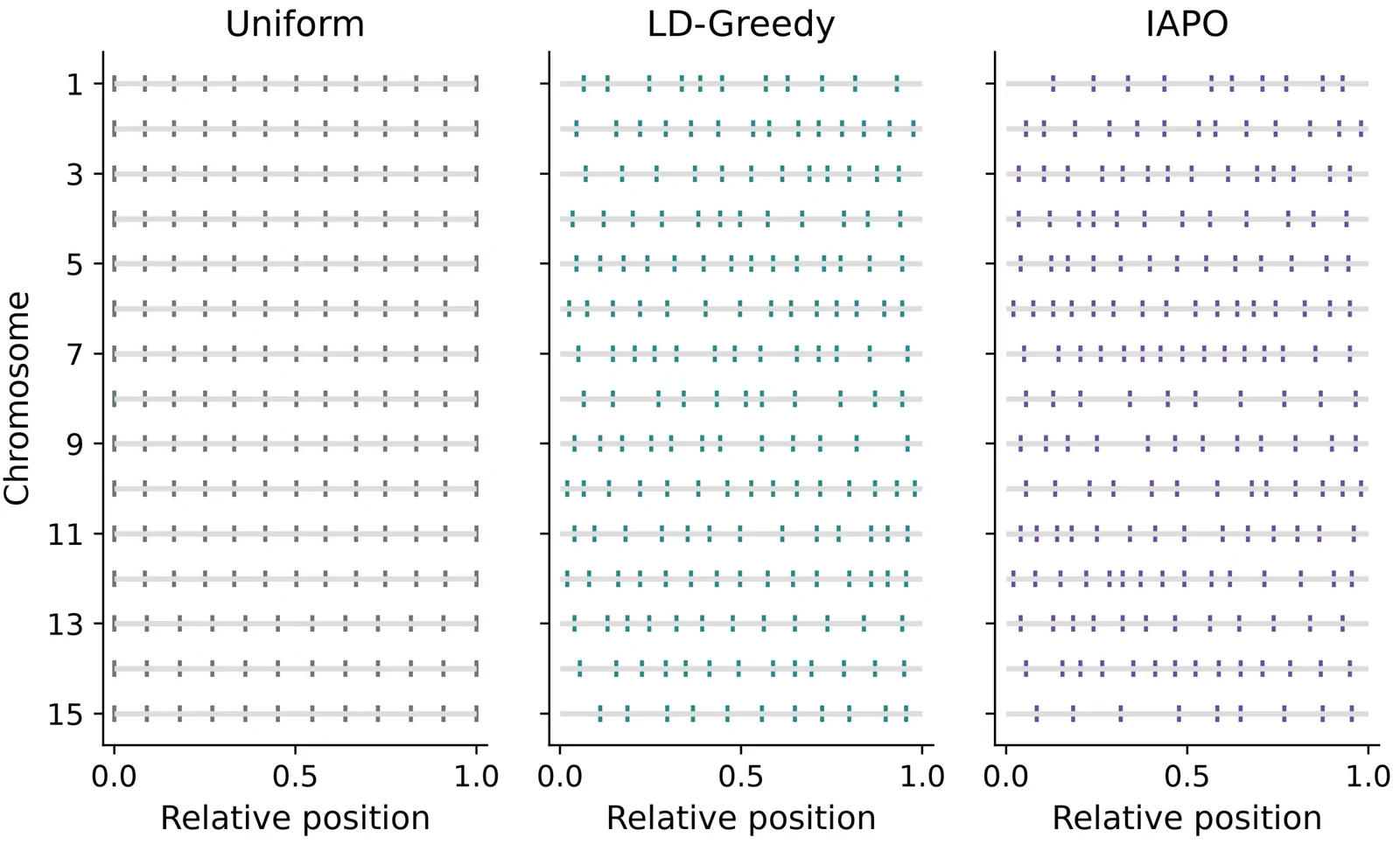
Actual 192-marker panels for one replicate. Each vertical tick identifies a selected locus on a normalized chromosome coordinate. Gray, teal, and purple ticks denote Uniform, LD-Greedy, and IAPO, respectively; light-gray horizontal lines indicate chromosomes.

Figure 3 compares marker locations selected by Uniform, LD-Greedy, and IAPO at k=192 for the first replicate (seed 20261001). The placements are computed from the archived selection indices. Uniform follows near-regular spacing; the greedy methods redistribute markers according to training scores. One placement plot is illustrative and does not summarize between-seed stability.

**Figure 4:**
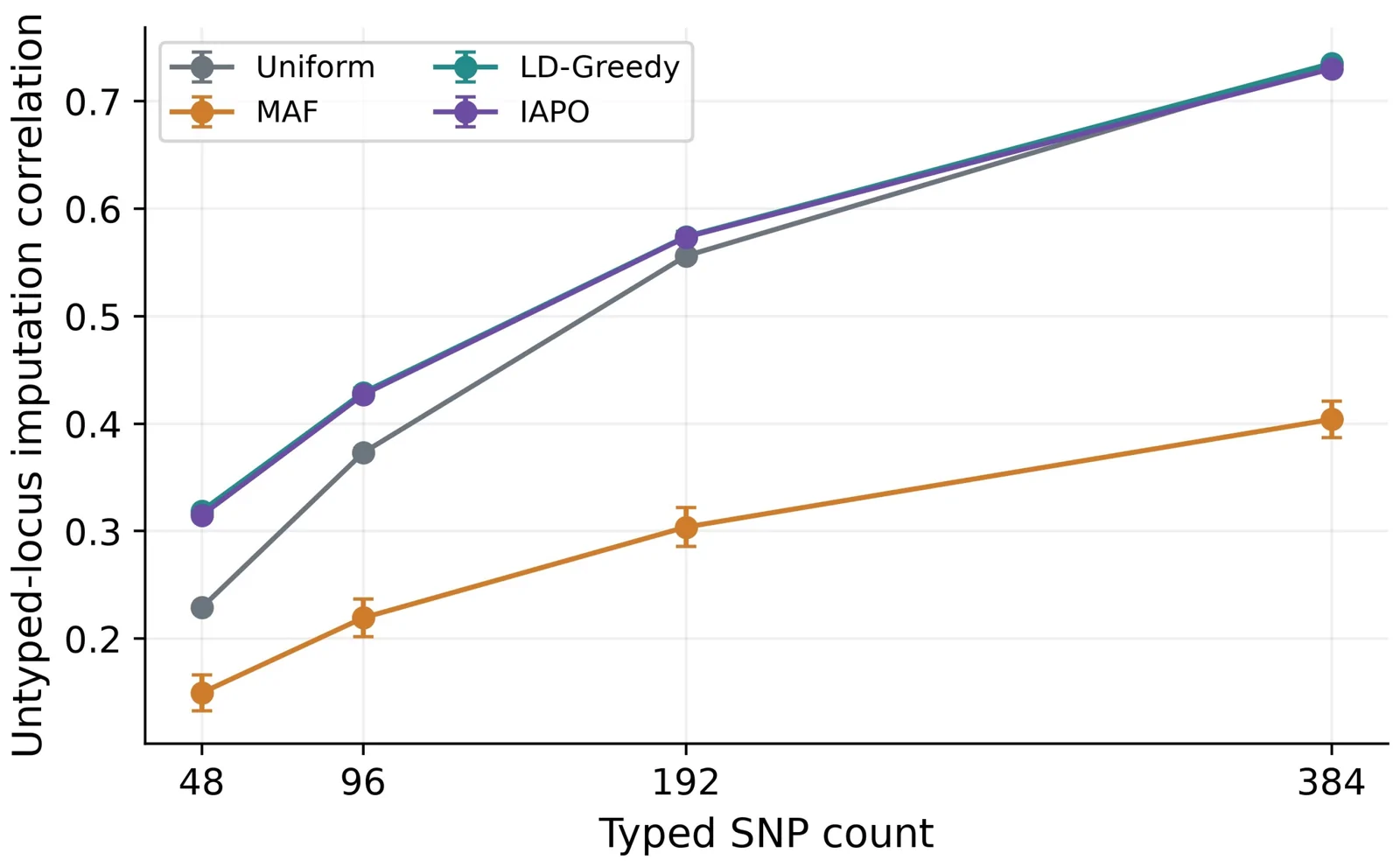
Untyped-locus imputation correlation. Points and error bars indicate means and SDs over ten independent seeds.

Figure 4 and Table 3 report repeated held-out imputation performance. At 192 markers, IAPO achieves 0.573±0.003, compared with 0.574±0.005 for LD-Greedy. Coverage-oriented placement improves reconstruction at very small budgets, but the utility term does not necessarily improve imputation. Correlation rises with density across the four methods; it must be interpreted alongside the downstream prediction results.

### 6.2. Prediction Accuracy and Cost Trade-Off

At 192 markers, IAPO gives r=0.347±0.092, LD-Greedy 0.289±0.070, and Uniform 0.217±0.105. The paired IAPO–LD-Greedy difference is 0.058 (95% CI 0.010–0.106); the IAPO–Uniform difference is 0.130 (0.040–0.220). These are exploratory paired comparisons across simulation seeds. Dense-marker ridge prediction gives 0.437±0.087, showing remaining information loss after sparse genotyping.

**Figure 5:**
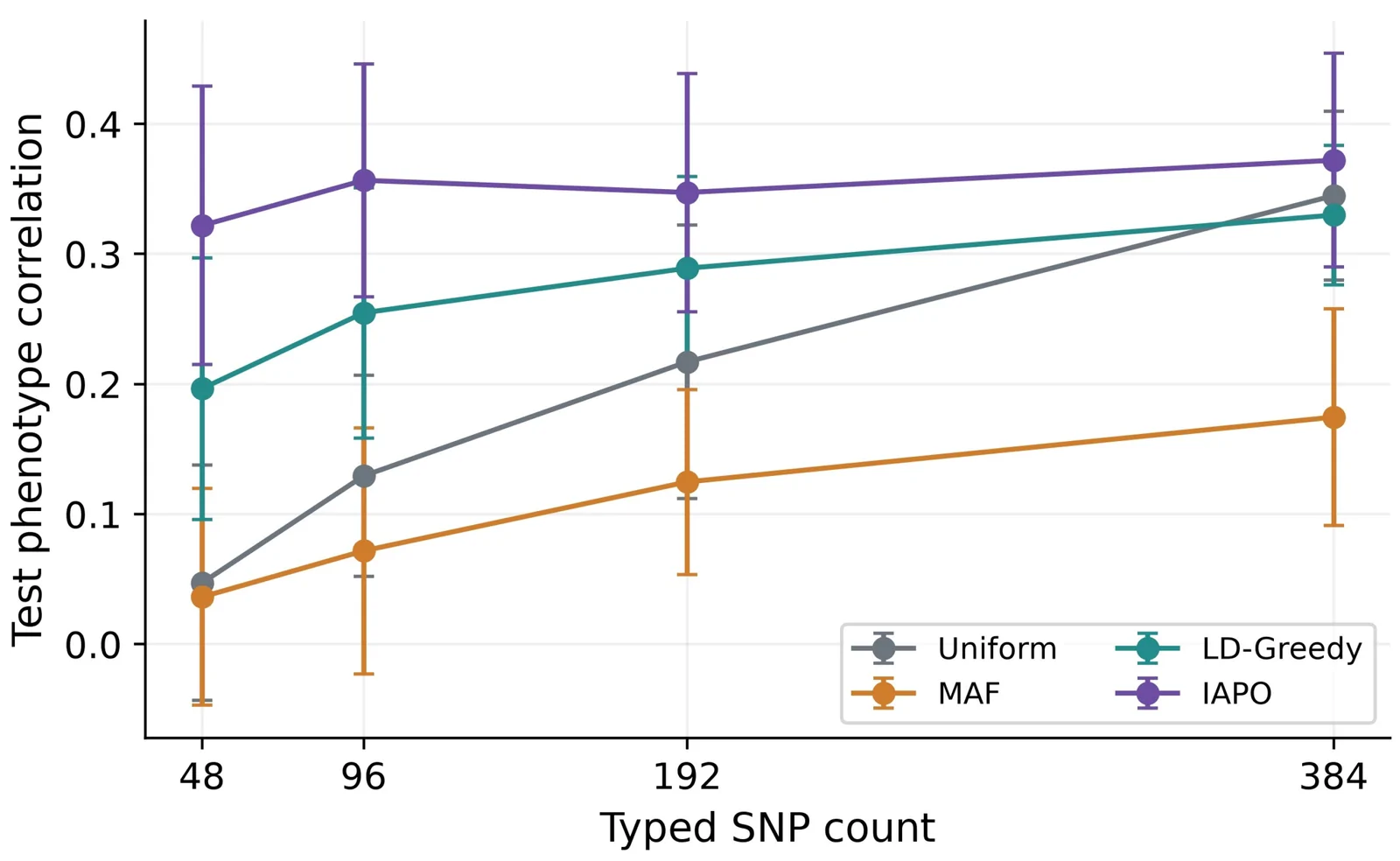
Test phenotype correlation by marker budget (mean ± SD over ten seeds). Panels and penalties are frozen before test evaluation.

Figure 5 does not support a unique optimum at 192 SNPs. IAPO means are 0.322, 0.357, 0.347, and 0.372 at 48, 96, 192, and 384 markers, respectively. The highest mean is at 384, while variation between seeds is substantial. At that budget, the paired difference from Uniform is 0.027 (95% CI −0.026–0.081). Thus, neither universal dominance nor a species-wide density recommendation follows.

**Figure 6:**
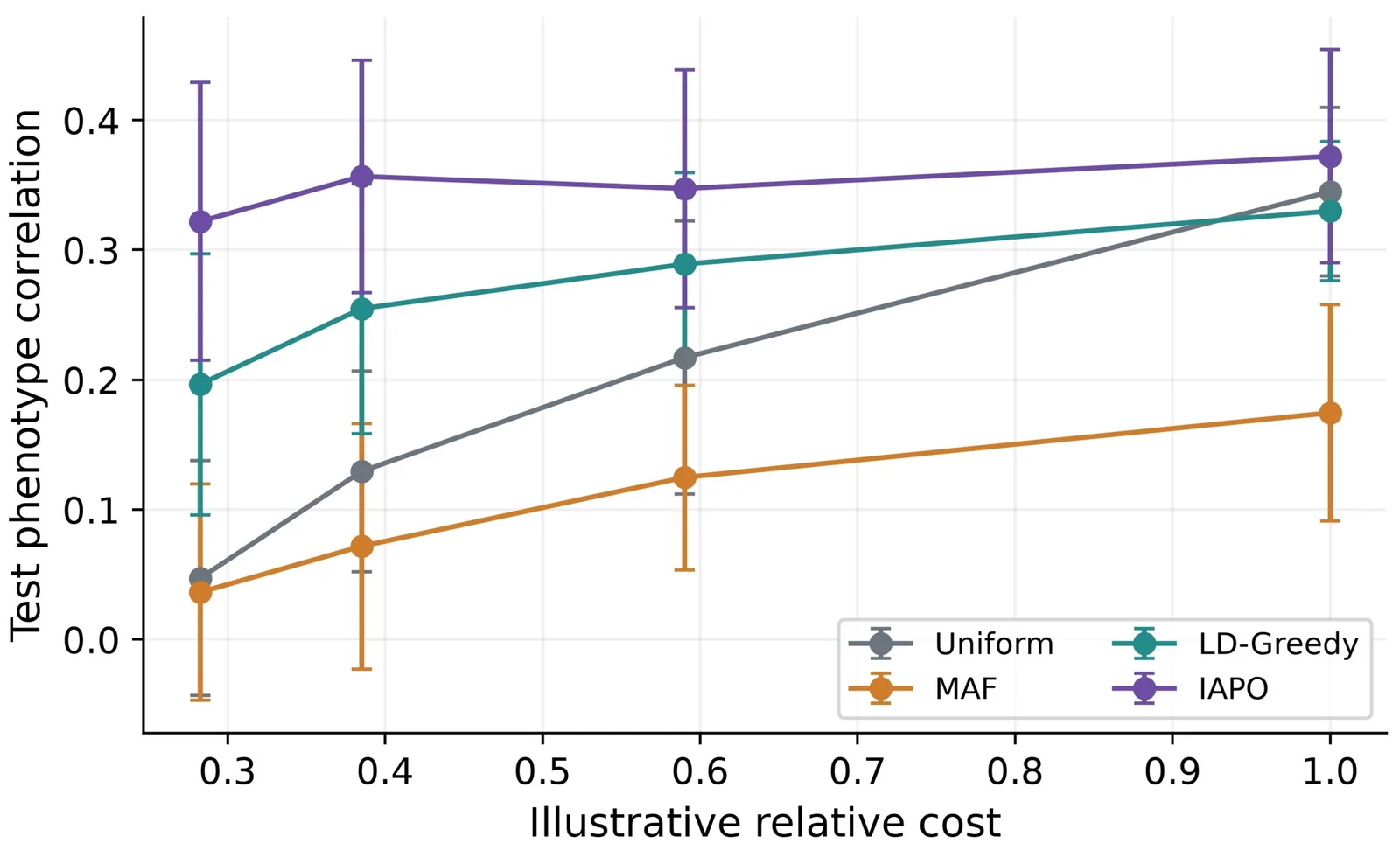
Prediction correlation versus the illustrative cost index (mean ± SD). The cost axis has no vendor-price interpretation.

Figure 6 combines the same predictions with Equation (37). The 192-marker cost index is 0.59, but its mean prediction accuracy is lower than that at 384 markers and also slightly lower than that at 96 markers. A practical budget must be chosen using validation performance and platform costs; maximizing a retrospectively observed test correlation would bias deployment decisions.

### 6.3. Candidate Ranking and Alternative Architectures

**Figure 7:**
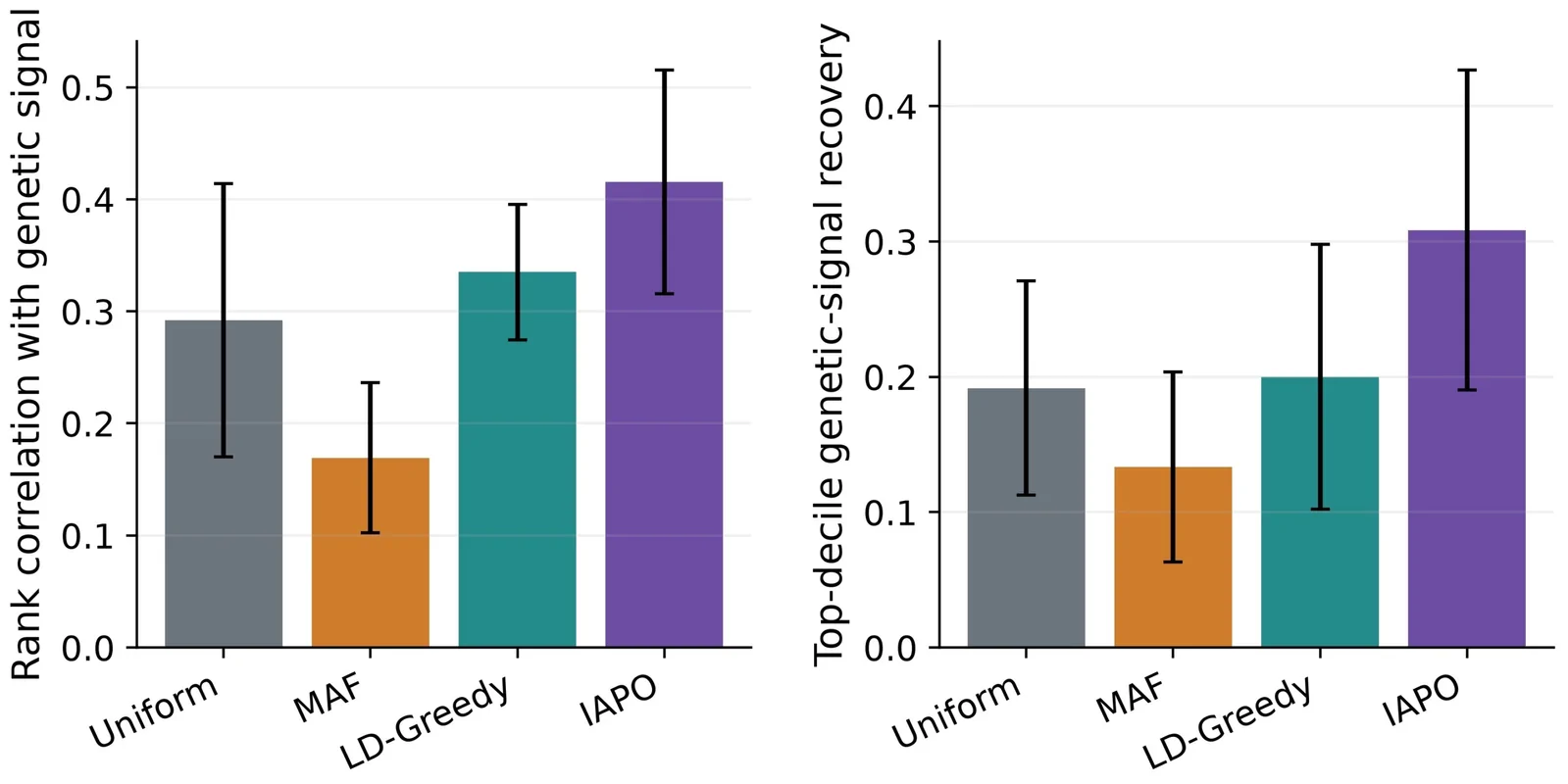
Ranking and top-decile recovery against the simulated genetic signal at 192 markers (mean ± SD). Gray, orange, teal, and purple bars denote Uniform, MAF, LD-Greedy, and IAPO, respectively. Black error bars indicate SD across ten independent seeds.

Table 4 and Figure 7 connect prediction to simulated candidate selection. At 192 markers, IAPO has genetic-signal rank correlation 0.415±0.100 and top-decile recovery 0.308±0.118. Top-decile recovery remains far below complete recovery. Phenotype and genetic-signal rankings are distinct because the phenotype contains noise; results for both thresholds and targets are retained in the archive. These outcomes are not realized genetic gain or empirical breeding-value accuracy.

The alternative architectures in Table 5 preserve IAPO’s higher mean correlation relative to the two displayed comparators, but performance changes with architecture. The IAPO mean is 0.425 for sparse additive effects, 0.339 for dense additive effects, 0.318 for stronger interactions, and 0.272 when the reference and target both have shorter LD. These checks broaden the simulation evidence without validating autotetraploid inheritance.

### 6.4. Imputer, Weight, and Population Sensitivity

The second imputer changes the comparison materially (Table 6). With reference nearest neighbors, IAPO and LD-Greedy prediction means are 0.235 and 0.234, respectively. Their near equality contrasts with the local-ridge difference. IAPO is therefore not shown to outperform coverage-only selection independently of imputation engine. At 192 markers, halving or doubling the default utility weight gives means of 0.333 and 0.397; removing chromosome balance gives 0.343. These diagnostic alternatives are not used to retune the reported default.

**Figure 8:**
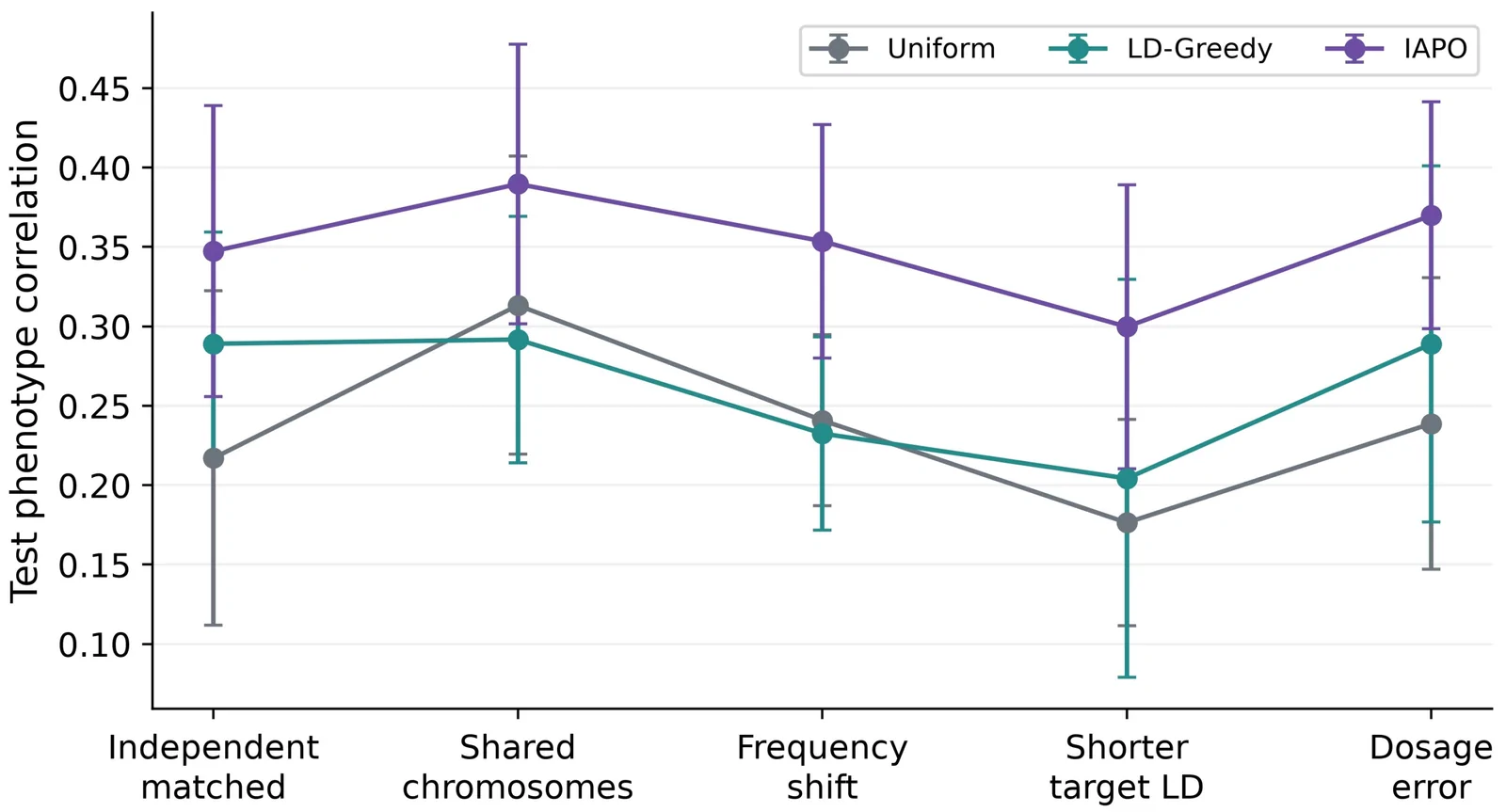
Distinct target-population conditions at 192 markers (mean ± SD). The horizontal axis lists scenarios, not an ordered severity scale. Dosage-error targets also have paired error-free controls in the archive.

Figure 8 shows IAPO prediction means of 0.347 for independently matched targets, 0.390 for chromosome-sharing targets, 0.354 after an allele-refresh shift, and 0.300 under shorter target LD. The dosage-error result is 0.370; its paired clean-control mean is 0.382, illustrating the measurement-error effect on the same individuals. Differences between independently generated target populations include sampling variation, so the curve is not evidence of monotone degradation. Shared chromosome segments are a relatedness proxy, not a pedigree simulation.

### 6.5. Coverage Assumption Diagnostic

For IAPO at 192 markers, the assumed locus-wise coverage inequality fails empirically at 49.3% of untyped loci in the matched condition, rising to 94.4% under shorter target LD. The matched mean dosage MSE is 0.549 versus a proxy envelope of 0.548; under shorter target LD, these values are 0.825 and 0.548. Thus, sample squared correlations cannot be treated as conservative recoverability bounds for this fitted imputer. Proposition 1 remains a conditional population statement; the optimization results are empirical evidence, not validation of its premise.

**Remark** **5.** *The revised benchmark separates three questions: approximation of the set objective, conditional imputation-error control, and observed prediction quality. The first is guaranteed by the stated assumptions, the second requires calibration that is not achieved by raw training correlations here, and the third varies with budget, imputer, and target population.*

### 6.6. Evaluation in the Published C. oleifera Family

Training-only quality filtering retained 5264–5289 markers across the ten fitting partitions. Table 7 compares the prespecified 192-marker panels with the dense baseline. For oil content, IAPO had a lower mean phenotype correlation than both Uniform and LD-Greedy; for fruit yield, its mean was higher than both. Predictive correlations were modest and split variation was substantial. These are held-out predictions of observed phenotypes, not correlations with known genetic values.

The paired mean IAPO minus LD-Greedy prediction-correlation differences at 192 markers were −0.049 for oil content and 0.046 for fruit yield. With the five-neighbor imputer, IAPO correlations were 0.188±0.181 and 0.187±0.124, respectively. These comparisons do not support a universal claim that the utility and balance terms improve prediction in a new biological dataset. The complete budget, ranking, error, and imputer results are retained in the empirical result tables, including unfavorable comparisons.

This reanalysis demonstrates that the workflow can ingest published biological genotypes, choose a sparse panel without using held-out phenotypes, reconstruct untyped marker states, and evaluate real traits. Its scope is narrower than field deployment. The observations come from one full-sib family, the marker states are not resolved polyploid dosages, and the outcome averages reuse historical years. Neither transfer to unrelated families nor future-year performance is established.

## 7. Discussion

### 7.1. Why Imputation-Aware Selection Matters

The main methodological implication is that ultra-low-density genotyping should be designed around the reconstruction step. A typed SNP is useful not only because it is polymorphic or associated with the current trait, but also because it anchors neighboring untyped variants. Equation (2) formalizes this idea without requiring a specific imputation engine. If a breeding program uses Beagle-like haplotype imputation, pedigree-aware imputation, or a learned multilocus model, $q_{ij}$ can be replaced by cross-validated recoverability measured under that engine. The submodularity proof still applies as long as the design uses non-negative fixed recoverability scores.

The results also clarify why maximizing imputation alone may be insufficient. In the synthetic benchmark at 192 markers, IAPO and LD-Greedy have similar imputation correlation but differ in prediction with the local ridge engine. That difference nearly disappears with the nearest-neighbor engine. A useful practical strategy is therefore to tune β/α jointly with panel size and imputer using nested validation, rather than treating the objective weights as universal constants. The present diagnostic weight comparisons demonstrate sensitivity and do not establish a preferred setting for a new trait.

### 7.2. Implications for Oiltea-Camellia Breeding

A deployment pathway for *C. oleifera* would begin with a dense reference population representing elite parents, key crossing families, and major production regions. Dense variants from genome resequencing or reduced-representation sequencing would be filtered for assay design, dosage reliability, and stable coordinates against the current reference genome. Cross-validated imputation recoverability would then be estimated within and across families. IAPO would select candidate panels at several budgets, after which the chosen SNPs would be converted to a targeted genotyping platform. Breeding seedlings could be assayed early, imputed back to the dense reference, and ranked by genomic best linear unbiased prediction (GBLUP) or another validated genomic predictor.

The approach can also coexist with marker-assisted selection. If validated large-effect loci for oil quality, fruit traits, flowering time, or stress response must always be present, they can be placed in a mandatory set *M* before greedy optimization. The residual budget is then applied to V\M, and the objective becomes F(M∪S)−F(M). Because submodularity is preserved under conditioning on a fixed set, the same greedy guarantee applies to the residual selection problem. This provides a clean mathematical mechanism for integrating trait-specific markers with genome-wide prediction markers.

### 7.3. Limitations and Validation Requirements

Three limitations are particularly important. First, the controlled benchmark is fully synthetic, whereas the empirical reanalysis uses normalized segregation states from one full-sib family. Neither component validates ploidy-resolved dosage inference or family-to-family transfer. Dosage support alone does not justify an autotetraploid population model. Double reduction changes segregation and identity-by-descent; preferential pairing changes linkage transmission; family-specific recombination changes local recoverability. Pedigree-based simulators such as AlphaSimR provide a route to explicitly modeling meiosis and autopolyploid inheritance [30]. These processes were not simulated here, and empirical haplotype calibration remains necessary.

Second, real resequencing introduces uncertain dosage calls, ambiguous read mapping, and structural variation [31]. A deployment study should retain genotype posterior dosages or likelihoods, exclude unreliable loci, estimate recoverability under family-separated cross-validation, and test a ploidy-appropriate haplotype or pedigree imputer. The added ±1 error experiment represents only a simplified measurement perturbation; it does not reproduce allele-specific calling bias. The theoretical submodularity result survives fixed non-negative scores, but the statistical interpretation must be recalibrated for the chosen engine and target population.

Third, the cost index is illustrative. Kompetitive allele-specific polymerase chain reaction (KASP) assays require locus-specific development and dosage-cluster validation, as illustrated in tetraploid potato [32]; targeted sequencing combines loci but introduces capture/amplicon balance, read-depth, multiplexing, and minimum-batch constraints. Assay failures can also change the effective panel. Consequently, a nominal 192-marker budget may map to different feasible sizes and per-sample prices on different platforms. No procurement recommendation is made.

These limitations do not negate the submodular guarantee or the deterministic ridge perturbation bound. They do limit biological interpretation. Before breeding use, the panel requires external validation across families, sites, years, and target traits, with empirical costs and realized selection outcomes. The simulation replicates describe the specified generators. The repeated empirical splits describe within-family prediction and cannot replace external biological replication.

**Remark** **6.** *The proposed framework is best viewed as a bridge between dense genomic discovery and routine breeding assays. Its theoretical value lies in converting panel design into an auditable optimization problem with diminishing returns and a downstream stability bound. Its practical value depends on extending the present within-family evaluation to representative reference populations, dosage-aware assays, and real genotyping costs. This division of labor keeps the method rigorous while making clear which conclusions are mathematical and which still require biological validation.*

## 8. Conclusions and Future Work

We developed IAPO, an imputation-aware framework for constructing ultra-low-density SNP panels, and evaluated it through synthetic tetraploid-dosage experiments and a separate reanalysis of published *C. oleifera* family data. The method combines recoverability of untyped loci, training-only predictive utility, and chromosome balance in a single monotone submodular objective. This structure yields a greedy 1−1/e approximation guarantee. A complementary perturbation theorem shows how genotype-imputation error propagates into ridge genomic prediction, providing a direct theoretical connection between panel design and downstream stability. Across ten seeds, the 192-marker local-ridge configuration improved mean phenotype correlation over coverage-only selection, but the advantage depended on imputation engine. Empirical prediction was modest, and IAPO had lower mean oil-content correlation than uniform placement at 192 markers with local ridge reconstruction. The within-family analysis does not establish a universally superior selector, an optimal 192-SNP budget, or transfer across breeding populations. Empirical calibration is essential because the raw recoverability proxy does not satisfy the conditional imputation bound in the benchmark.

Future work will proceed in three directions. First, the objective should be recalibrated on multi-family and multi-environment *C. oleifera* reference populations with experimentally observed dosage uncertainty, haplotypes, and recombination. Second, robust panel design should optimize across traits and breeding cycles simultaneously, including mandatory major-effect markers and worst-case or distributionally robust recoverability. Third, prospective field validation should compare the selected ultra-low-density panel with dense sequencing in independent seedlings and mature trees, measuring not only prediction correlation but also top-candidate recovery, realized genetic gain, assay failure, and full per-sample cost. 

## Figures and Tables

**Table 1:** Lazy greedy IAPO panel construction.

**Require:** Candidate set *V*, recoverability matrix *Q*, utilities $u_{i}$, chromosome map c(i), and budget *k*.	
**Step**	**Procedure**
1	S←⌀; initialize locus coverage and chromosome counts to zero.
2	Compute initial upper bounds for all marginal gains and store them in a max-priority queue.
3	**while** |S|<k **do**
4	Pop the candidate *e* with the largest stored upper bound.
5	Recompute $\Delta_{F}\left(e|S\right)$ using current coverage and chromosome count.
6	**if** the recomputed gain remains maximal **then**
7	S←S∪{e}; update coverage and chromosome count.
8	**else** return the recomputed gain to the priority queue.
9	**end if**
10	**end while**; **return** *S*.

**Table 2:** Synthetic benchmark configuration and repeated evaluation.

Setting	Value
Individuals	720
Training/validation/test	480/120/120
Chromosomes	15
Dense SNPs	3000
Ploidy and dosage	tetraploid, 0–4
Additive QTLs	72
Weak epistatic pairs	18
Genetic-signal fraction	0.55
Panel sizes	48, 96, 192, 384 SNPs
Ridge λ grid	5, 15, 40, 100, 250, 600
Independent replicate seeds	20261001–20261010

**Table 3:** Repeated matched-population performance (mean ± SD, ten seeds). Imputation metrics exclude typed loci.

Method	SNPs	Imputation *r*	Dosage NRMSE	Prediction *r*	Prediction RMSE
Uniform	48	0.229±0.003	0.221±0.001	0.047±0.090	1.056±0.070
MAF	48	0.150±0.017	0.224±0.001	0.036±0.083	1.033±0.077
LD-Greedy	48	0.319±0.002	0.215±0.001	0.196±0.100	1.009±0.080
IAPO	48	0.315±0.002	0.215±0.001	0.322±0.107	0.971±0.066
Uniform	96	0.373±0.004	0.210±0.001	0.129±0.077	1.068±0.076
MAF	96	0.219±0.018	0.220±0.001	0.072±0.095	1.054±0.087
LD-Greedy	96	0.429±0.004	0.205±0.001	0.255±0.096	1.013±0.070
IAPO	96	0.427±0.003	0.205±0.000	0.357±0.090	0.975±0.069
Uniform	192	0.556±0.003	0.189±0.001	0.217±0.105	1.099±0.077
MAF	192	0.304±0.018	0.214±0.001	0.125±0.071	1.065±0.070
LD-Greedy	192	0.574±0.005	0.185±0.001	0.289±0.070	1.059±0.075
IAPO	192	0.573±0.003	0.185±0.000	0.347±0.092	1.007±0.059
Uniform	384	0.735±0.003	0.154±0.001	0.345±0.065	1.040±0.082
MAF	384	0.404±0.017	0.204±0.002	0.175±0.083	1.088±0.045
LD-Greedy	384	0.735±0.003	0.154±0.001	0.330±0.053	1.064±0.066
IAPO	384	0.730±0.003	0.154±0.001	0.372±0.082	1.005±0.069

**Table 4:** Candidate ranking at 192 markers (mean ± SD, ten seeds). Genetic signal denotes the known simulated total signal.

Method	ρ Phenotype	ρ Genetic	Top 5% Genetic	Top 10% Genetic
Uniform	0.211±0.100	0.292±0.122	0.183±0.123	0.192±0.079
MAF	0.111±0.079	0.169±0.067	0.117±0.112	0.133±0.070
LD-Greedy	0.265±0.080	0.335±0.061	0.167±0.079	0.200±0.098
IAPO	0.329±0.110	0.415±0.100	0.200±0.172	0.308±0.118

**Table 5:** Architecture and reference sensitivity at 192 markers (prediction *r*, mean ± SD). Each condition uses ten seeds.

Condition	Uniform	LD-Greedy	IAPO
72 QTLs, weak interactions	0.217±0.105	0.289±0.070	0.347±0.092
12 additive QTLs	0.312±0.107	0.274±0.089	0.425±0.122
300 additive QTLs	0.264±0.088	0.241±0.130	0.339±0.089
72 QTLs, stronger interactions	0.197±0.088	0.272±0.068	0.318±0.106
Short-LD reference and target	0.132±0.088	0.168±0.077	0.272±0.100

**Table 6:** Imputation-engine comparison at 192 markers (mean ± SD, ten seeds). kNN denotes *k*-nearest-neighbor imputation using reference individuals.

Method	Local Ridge Prediction *r*	kNN Prediction *r*	kNN Imputation *r*
Uniform	0.217±0.105	0.091±0.107	0.350±0.004
LD-Greedy	0.289±0.070	0.234±0.083	0.374±0.006
IAPO	0.347±0.092	0.235±0.081	0.372±0.006

**Table 7:** Empirical within-family performance at 192 markers and with dense markers. Values are means ± SD over two repetitions of five-fold cross-validation of 164 offspring. RMSE uses fitting-standardized phenotypes; imputation *r* excludes typed and originally missing test calls. SDs describe overlapping split variation, not independent population replication.

Trait	Method	Markers	Prediction *r*	Prediction RMSE	Imputation *r*
Oil content	Uniform	192	0.210±0.206	1.164±0.207	0.130±0.004
Oil content	Diversity	192	0.063±0.140	1.278±0.188	0.123±0.010
Oil content	LD-Greedy	192	0.117±0.160	1.204±0.210	0.265±0.004
Oil content	IAPO	192	0.067±0.112	1.179±0.178	0.261±0.007
Oil content	Dense	5264–5289	0.189±0.174	1.000±0.207	—
Fruit yield	Uniform	192	−0.047±0.132	1.342±0.185	0.130±0.004
Fruit yield	Diversity	192	0.014±0.220	1.279±0.209	0.123±0.010
Fruit yield	LD-Greedy	192	0.107±0.108	1.227±0.077	0.265±0.004
Fruit yield	IAPO	192	0.153±0.145	1.143±0.138	0.262±0.005
Fruit yield	Dense	5264–5289	0.116±0.125	1.029±0.143	—

## Data Availability

The complete executable code, data, and results are publicly archived in Zenodo (version 2.0.0; DOI: https://doi.org/10.5281/zenodo.23553690; repository record: https://zenodo.org/records/23553690 (accessed on 22 October 2026)) [33]. The Code_Data_Results_R2.zip archive (Supplementary Materials) contains all 50 synthetic condition-by-seed datasets, the empirical source and derived inputs, sample eligibility and train/validation/test splits, selected panels, individual predictions, numerical summaries, figure scripts, dependency versions, verification scripts, and a SHA-256 file manifest. The empirical source is the open Supplementary Materials of Lin et al. [2] (https://doi.org/10.3390/ijms25168840); the unmodified source ZIP is redistributed with its original attribution and CC BY 4.0 license. The original code, simulated data, derived results, and documentation in this release are also licensed under CC BY 4.0. No new field data were collected.

## Supplementary Materials

The following supporting information can be downloaded at https://www.mdpi.com/article/10.3390/plants15213346/s1, Supplementary File S1. Code, Data and Results archive (Code_Data_Results_R2.zip, version 2.0.0), containing executable Python code; 50 synthetic condition-by-seed datasets; the original published *Camellia oleifera* family source data and derived analysis inputs; sample eligibility records and data partitions; selected SNP panels and individual predictions; numerical summaries and verification records; scripts and source images for Figure 1, Figure 2, Figure 3, Figure 4, Figure 5, Figure 6, Figure 7 and Figure 8; dependency specifications, attribution, licensing, and a SHA-256 file manifest.
